# Evidence on the preparedness and practice needs of the home care workforce to support older LGBTQ+ people: a rapid review protocol

**DOI:** 10.1136/bmjopen-2025-110207

**Published:** 2026-02-02

**Authors:** Jolie R Keemink, Willem J Stander, Benjamin Thomas, Paul Willis

**Affiliations:** 1 Centre for Health Services Studies, School of Social Sciences, University of Kent, Canterbury, UK; 2 Institute for Mental Health, School of Psychology, University of Birmingham, Birmingham, England, UK; 3 School of Nursing and Midwifery, London South Bank University, London, UK; 4 Centre for Adult Social Care Research, School of Social Sciences, Cardiff University, Cardiff, UK

**Keywords:** Sexual and Gender Minorities, Aged, Social Support, Health Workforce

## Abstract

**Abstract:**

**Introduction:**

Older people who identify as lesbian, gay, bisexual, trans, queer or other marginalised sexualities and gender identities (LGBTQ+) still face significant barriers and inequalities when accessing adult social care services. Little is known about the preparedness of the care workforce to support older LGBTQ+ individuals, particularly within home care services. While a few previous reviews have examined the perspectives of older LGBTQ+ people on the preparedness of the home care workforce, none have included the perspectives of the workforce itself or compared both perspectives. This is a protocol for a rapid review that aims to explore what is known about the preparedness and practice needs of the home care workforce to support older LGBTQ+ people, with a particular focus on workforce perspectives.

**Methods and analysis:**

A rapid review method was selected to expedite the review process to support further study development and dissemination. Two electronic databases, SCOPUS and Web of Science, will be searched, as well as six subject-specific databases, including Social Care Institute for Excellence, Skills for Care, Social Care Wales, Homecare Association, Stonewall UK, LGBT Foundation UK and SAGE US. There are no search date restrictions. Study quality will be assessed using the Quality Assessment with Diverse Studies tool and the Grading of Recommendations, Assessment, Development and Evaluations considerations will be used to consider certainty of evidence. Data will be synthesised using narrative synthesis, including a descriptive summary of included studies and their methodological quality. All preferred reporting items for review protocols have been included, as recorded by the Preferred Reporting Items for Systematic Reviews and Meta-Analyses Protocol.

**Ethics and dissemination:**

Ethical approval is not required for the protocol and review. Manuscripts for the protocol and completed review will be submitted to a peer-reviewed journal, and findings will be shared in webinars for the home care workforce and at academic conferences.

**PROSPERO registration number:**

CRD420251038242.

**STRENGTHS AND LIMITATIONS OF THIS STUDY**

- By transparently reporting on the proposed methodology for this rapid review, we mitigate the risks of bias potentially involved in rapid methods.
- An older LGBTQ+ (lesbian, gay, bisexual, trans, queer or other marginalised sexualities and gender identities) person contributed to the funding application for the project, which included the proposed question, definition of key concepts and method for this rapid review protocol.
- A limitation of our rapid review methodology is that only studies published in English will be included.

## Introduction

 Despite advancements in equality legislation, many older lesbian, gay, bisexual, trans, queer or other marginalised sexualities and gender identities (LGBTQ+) people still face significant barriers and inequalities when accessing adult social care services across the globe.^1^,^9^ In the current study, older is defined as aged 60 and over, after consultation with the study’s public advisory group. Adult social care (also referred to as long-term care) provides support to people over 18 who need support with daily living and includes (but is not limited to) services such as residential care (care homes and nursing homes), day care centres, extra-care housing, home-based care and reablement services.^10^ A substantial number of social care providers remain unaware of the relevance of sexuality and gender in relation to care and may hold discriminatory assumptions rooted in ageism, heteronormativity and cisnormativity (the assumption that cisgender people, whose sex assigned at birth corresponds with their gender identity, are the norm).^1 11 12^ Consequently, older LGBTQ+ individuals and their care needs and life experiences are often neglected.^12^,^15^ At the same time, the older LGBTQ+ population is more likely to require support from social care services due to a range of disproportionate health inequalities, a greater likelihood of living alone and a lack of informal social support from biological children or family members traditionally relied on for caregiving.^5 16 17^ As a result, many older LGBTQ+ people are uncomfortable disclosing their LGBTQ+ identity, hesitant to access services and have poorer health outcomes.^18^ These health inequalities are further exacerbated by minority stress, which often stems from a lifetime of discrimination, social exclusion and institutional erasure, as well as the historical criminalisation and pathologisation of LGBTQ+ identities.^19^

This is an important issue to address, as data suggest that in many countries a substantial number of older people identify as LGBTQ+, and this is likely to increase in the future. Data are all estimates and vary from source to source, as systematic data collection on LGBTQ+ status is lacking worldwide, but it is suggested that 1 in 100 older people identify as LGBTQ+.^20^ This includes 120 000–1 million people in the UK,^21^,^23^ 3 million in the USA^24 25^ and 53 000 in Australia.^26^ Research on older LGBTQ+ people’s experiences of care and workforce preparedness to support this population has thus far focused primarily on residential care, extra-care housing or healthcare, with several studies aiming to improve inclusive provision.^79 27^,^30^ However, less is known on the state of LGBTQ+ inclusion in home care services, particularly in the UK, where the research team is based.^31^ It is unclear how prepared the home care workforce is to provide LGBTQ+ inclusive care. In particular, there appears to be a very limited understanding of the views and experiences of the home care workforce when it comes to their preparedness and needs in relation to supporting older LGBTQ+ people. By preparedness, we mean whether the workforce has the skills, knowledge and confidence to support the LGBTQ+ population. It is important to address this knowledge gap to ensure that older LGBTQ+ people’s experiences of home care are recognised, workforce experiences of supporting older LGBTQ+ people are acknowledged, and the home care workforce is adequately skilled to provide affirmative and inclusive care.

This need is underscored by a broader policy shift towards increased home-based care provision both in the UK^32^,^34^ and internationally.^35^,^38^ Although care at home may foster independence, inviting someone into one’s home can be experienced as a disruption to the private space.^17^ This is particularly relevant for older LGBTQ+ people, who are known to experience interpersonal and social pressures to conceal their identity when it comes to housing and care^5 16 39^ and whose home may be one of the few places where they can safely express themselves. Research also shows that a positive experience of home care is associated with improved quality of life,^40^ further illustrating the importance of developing provision that is safe and inclusive for everyone.

There are currently no reviews examining the perspective of the home care workforce on their preparedness to support older LGBTQ+ people, only reviews that either focus exclusively on US-based evidence, examine a different segment of the care workforce or consider only the perspectives of older LGBTQ+ people.^141^,^43^ A comprehensive understanding of the available evidence about the current preparedness and practice needs of the home care workforce is essential to identify knowledge and skill gaps and inform the development of LGBTQ+ inclusive home care. This knowledge will be essential for policymakers and care sector support organisations to understand how to best support the workforce in developing their inclusive practice. This paper presents the protocol for a rapid review with the primary aim to synthesise existing evidence on the preparedness and practice needs of the home care workforce to support older LGBTQ+ people, with a particular focus on identifying workforce perspectives.

## Methods and analysis

Rapid reviews of the literature offer the advantage of time efficiency compared with systematic reviews by streamlining and accelerating the review process while maintaining methodological rigour.^44^ The Cochrane recommendations for rapid reviews^45^ recommend several areas where the review process can be streamlined, including limiting the number of outcomes, search databases and reviewers. The method can be used to rapidly identify gaps in existing evidence in situations where the need for evidence is time-bound. It is important to acknowledge that the rapid review method may also hold limitations; rapidity may increase the risk of missing evidence or errors in synthesis and quality assessment.^46 47^ In an effort to minimise these risks, we publish this peer-reviewed protocol, as well as follow clear guidelines and recommendations, such as Preferred Reporting Items for Systematic Reviews and Meta-Analyses (PRISMA) and Cochrane. The current review is part of a larger funded study examining LGBTQ+ inclusion in home care and serves as a foundation for later stages in the project. Rapid collection of evidence will ensure the project addresses the most prominent evidence gaps effectively and that review findings can be shared with the home care workforce, policymakers and sector support organisations in a timely manner.

Following the PRISMA 2020 guidelines^48^ and interim guidance for rapid reviews (PRISMA-RR),^49^ this protocol has been registered on PROSPERO (CRD420251038242), the international prospective register of systematic reviews. We followed the PRISMA Protocols (PRISMA-P)^50^ in the write-up of this protocol. The completed PRISMA-P checklist can be found in online supplemental appendix 1. We will adhere to the guidelines set out in the interim guidance (PRISMA-RR), as well as the Cochrane recommendations for rapid reviews, when conducting and reporting on the review.

### PICO statement

The main research question the review will answer is: What is known about the preparedness and practice needs of the home care workforce to support older LGBTQ+ people? The populations (P) under examination are the home care workforce and older LGBTQ+ people. The home care workforce includes any type of role involved in home care provision (management and frontline). The older LGBTQ+ population includes any person over 60 years of age identifying as LGBTQ+. The intervention (I) is home care, which we define as the frontline delivery of social care in people’s own homes, including personal care (support with personal hygiene and dressing) and reablement services (care after illness or hospital discharge). There is no comparison (C) measure in this study. The outcomes (O) we are interested in are the preparedness and practice needs of the home care workforce to care for older people. We define preparedness as the skills, knowledge and confidence to provide LGBTQ+ affirmative care. Practice needs are defined in relation to the lack of skills, knowledge and confidence. The identification of these outcomes will help the understanding of the current state of LGBTQ+ inclusion in home care and how it can be improved. Based on previous research on other care settings, it is anticipated that preparedness will be limited.^45 7^,^9^

### Eligibility criteria

Studies are deemed eligible for inclusion if they examine the preparedness and practice needs of the home care workforce to support older LGBTQ+ people. Studies concerning home care alongside other types of social care delivery will be included, but studies exclusively examining other types of social care delivery (eg, care homes, nursing homes, supported living, day care centres and hospice care) will be excluded. Studies must concern care for people over 60 years identifying as LGBTQ+. Studies pertaining to care for the LGBTQ+ population across the lifespan will be considered if care for older people is included. Studies exclusively considering care for LGBTQ+ people under 60 years will be excluded. Studies concerning only specific sexual orientations or gender identities (eg only gay men) will be included. We are particularly interested in studies examining preparedness and practice needs from the perspective of the home care workforce but will also include evidence from the perspective of older LGBTQ+ people and their partners or informal carers to maximise the evidence captured by the review and to compare findings from different perspectives. While perspectives of older LGBTQ+ people have been captured in previous reviews,^141^,^43^ there are no reviews examining perspectives of the home care workforce or reviews comparing different perspectives.

All study designs will be considered, including any review type (eg systematic, realist, scoping and meta-analysis), observational studies, cross-sectional studies, qualitative interview studies and randomised control trials. Only published documents will be considered. The main outcomes for which data will be sought are the preparedness and practice needs of the home care workforce to care for older LGBTQ+ people. We will include studies measuring and examining these outcomes quantitatively (eg using scales in a survey) or qualitatively (eg using interviews). Studies can be from any country, but the publication language must be English to be included, as recommended for rapid reviews by the Cochrane recommendations.^45^ This may pose a limitation to the completeness of the included evidence, but research suggests that excluding non-English publications does not significantly change the conclusions of reviews.^51^ Although this is a rapid review, and the Cochrane recommendations allow for the exclusion of grey literature to accelerate the review process, we will also consider grey literature. Based on the authors’ expertise in this area, it is expected that there will be a scarcity of literature, and therefore, all available material will be considered, both academic and grey literature. We argue that this is particularly important for topics such as LGBTQ+ inclusion for older people, which until recently has been neglected in research.^52 53^ Furthermore, no previous review has included grey literature databases. Similarly, we will consider studies of any methodological quality, but the quality of evidence will be reported in line with Cochrane recommendations.

### Identification and selection

The search will be carried out between August and November 2025. The final search strategy will be developed by two members of the research team with previous experience of planning, conducting and publishing scoping and realist reviews, and advice from the university librarian will be sought to finalise the search strategy. The draft search strategy for the academic literature can be found in table 1.

**Table 1:** A draft search strategy for the academic literature

Concept	Search terms
Preparedness	prepared* OR comfort* OR training OR readiness OR competence OR “cultural competence” OR knowledge OR attitude* OR skill* OR confidence OR perception* OR awareness OR abilit* OR capabilit*
AND	
Home care	“home* care” OR homecare OR “domiciliary care” OR “personal care” OR “social care” OR “adult social care” OR reablement OR “long term care” OR “home service*” OR “community care” OR “home help” OR “in-home care” OR “home assistance” OR “home healthcare” OR “care at home”
AND	
LGBTQ+	LGB* OR “sexual minorit*” OR “sexual orientation” OR “gender identit*” OR “gender minorit*” OR “sexual minorit*” OR gay* OR bisexual* OR lesbian* OR trans* OR queer* OR asexual* OR non-heterosexual* OR “marginali* sexualit*” OR “marginali* gender*”
AND	
Older people	“older adult*” OR “older people” OR elder* OR senior* OR “older person*” OR ageing OR ag ing OR “senior citizen*” OR geriatrics OR retired OR aged OR older

We will use relevant Medical Subject Headings, truncate as appropriate, and use variant spellings (asterisks are ‘wild’ operators). Search terms must be present in the title or abstract of the identified literature. Following Cochrane recommendations of limiting the number of search databases for a rapid review, two bibliographical databases will be searched (SCOPUS and Web of Science). Reference lists of related reviews will be searched for missed references. We will also search subject-specific databases that could be viewed as sources of grey literature (Social Care Institute for Excellence, Skills for Care, Social Care Wales, Homecare Association, Stonewall UK, LGBT Foundation UK and SAGE US). These organisations are leaders in advocacy and service provision for LGBTQ+ populations or are key workforce support organisations, with established roles in producing and disseminating research, policy reports and community-based evidence on LGBTQ+ inclusion in care. Including both UK and US sources allows for broader contextual insights. As these databases do not support the above search strategy, we will use an adapted strategy, inserting relevant terminology in the search field on the respective websites. There are no search date restrictions.

Search results will be downloaded to Zotero,^54^ a reference management software programme and screened for duplicates, which will be removed. The remaining articles will be uploaded to Rayyan for abstract screening and document selection. As per Cochrane recommendations, all abstracts will be screened by one reviewer, and a second reviewer will screen all excluded abstracts. Full texts of eligible articles will be independently screened against the eligibility criteria by one reviewer, with a second reviewer screening all excluded documents. Reasons for exclusion will be noted. Any disagreements will be solved with a third reviewer. We will create a PRISMA-compliant flowchart to record the steps of the identification and selection process.

### Data extraction and management

A spreadsheet for data extraction will be created in Microsoft Excel and piloted using three documents to allow for refinements. As per recommendations, one reviewer will independently extract data from all included documents, with a second reviewer verifying all entries. The extraction table will include information such as authors, title, year, country, study design, setting, sample, outcome measures related to preparedness and practice needs of the home care workforce, and key findings. Reviewers will meet to compare and discuss extraction results and to resolve any discrepancies. A combined spreadsheet will be created for data synthesis.

### Quality assessment

Cochrane recommends assessing included studies for risk of bias. As described above, to ensure all available literature in this scarcely researched area is considered, we will include studies regardless of their methodological rigour. However, for transparency and interpretation of review findings, we will assess and report the quality of the included studies, using the quality assessment with diverse studies tool.^55^ This assessment tool is particularly suitable for reviews including diverse study designs and has been successfully used by one of the authors of this review.^56^ The tool requires a rating from 0 to 3 across 13 domains: theory, aims, setting/population, design, sampling, rationale and appropriateness of data collection tools, procedure, recruitment data, rationale and appropriateness of analysis methods, stakeholder engagement and limitations. One reviewer will independently assess all included studies for quality, with a second reviewer verifying all assessments. Disagreements will be resolved with a third reviewer if necessary.

### Data analysis and synthesis

Data recorded in the extraction table will be summarised and interpreted by two reviewers. Based on previous, related reviews,^141^,^43^ it is expected that we will identify both qualitative and quantitative studies. These data will be analysed using thematic analysis following the approach for the thematic synthesis of qualitative data in reviews set out by Thomas and Harden^57^ as well as aligning with the narrative, configurative approach detailed by Gough and colleagues.^58^ The authors suggest this method helps ensure a transparent connection between review findings and the primary studies. Quantitative data will be reported following the synthesis without meta-analysis reporting guidelines.^59^ Summary tables will be used to present both qualitative and quantitative data. Cochrane guidelines also recommend assessing certainty of evidence during data synthesis using the five Grading of Recommendations, Assessment, Development and Evaluations considerations.^60^ We will use this to assess the certainty of the identified body of evidence and report the outcome of the assessment in the review.

### Public involvement

Cochrane recommendations for rapid reviews encourage the direct involvement of key stakeholders. An older LGBTQ+ person contributed to the funding application for the project, which included the proposed question, definition of key concepts and method for this rapid review. The project’s public advisory group, including older LGBTQ+ people (who are also co-researchers on the project), will be consulted from September onwards (when the first meeting is taking place) and will be able to input from the stage of emerging findings.

## Ethics and dissemination

Ethical approval will not be required for the review protocol and the rapid review itself. In addition to the manuscript for this protocol, a manuscript of the review will be submitted to a peer-reviewed academic journal. We will share the findings and implications of the review in practice-focused webinars (aimed at the home care workforce) organised in collaboration with key stakeholder organisations in adult social care and at academic conferences. Findings of the review will also be informative for the subsequent phases of the wider study on LGBTQ+ inclusion in home care involving primary data collection. We will also develop an easy-read summary of the findings with support from the study’s public advisory group.

## Supplementary material

10.1136/bmjopen-2025-110207 online supplemental file 1

## References

[1] Caceres BA, Travers J, Primiano JE (2020). Provider and LGBT Individuals’ Perspectives on LGBT Issues in Long-Term Care: A Systematic Review. Gerontologist.

[2] Hong H (2024). Study on Long-Term Care Service Awareness, Needs, and Usage Intention of Older Adult Male Homosexuals in Taiwan and Their Ideal Long-Term Care Service Model. Healthcare.

[3] Löf J, Olaison A (2020). ‘I don’t want to go back into the closet just because I need care’: recognition of older LGBTQ adults in relation to future care needs. Eur. J. Soc. Work.

[4] King A, Stoneman P (2017). Understanding SAFE Housing – putting older LGBT* people’s concerns, preferences and experiences of housing in England in a sociological context. HCS.

[5] Kneale D, Henley J, Thomas J (2021). Inequalities in older LGBT people’s health and care needs in the United Kingdom: a systematic scoping review. Ageing Soc.

[6] Williams KA, Dakin EK, Lipschutz A (2022). LGBT+ Older Adults in Rural South Central Appalachia: Perceptions of Current and Future Formal Service Needs. J Gerontol Soc Work.

[7] Willis P, Maegusuku-hewett T, Raithby M (2016). Swimming upstream: the provision of inclusive care to older lesbian, gay and bisexual (LGB) adults in residential and nursing environments in Wales. Ageing Soc.

[8] Willis P, Raithby M, Dobbs C (2021). ‘I’m going to live my life for me’: trans ageing, care, and older trans and gender non-conforming adults’ expectations of and concerns for later life. Ageing Soc.

[9] Willis P, Beach B, Powell J (2023). “There isn’t anybody else like me around here”: the insider-outsider status of LGBT residents in housing with care schemes for older people. *Front Sociol*.

[10] The King’s Fund (2025). Key facts and figures about adult social care. https://www.kingsfund.org.uk/insight-and-analysis/data-and-charts/key-facts-figures-adult-social-care.

[11] McMullen-Roach S, Kumar S, Inacio M (2025). The Perspectives and Experiences of Older LGBTI+ Adults About Long-Term Care: A Qualitative Systematic Review and Meta-Synthesis. Gerontologist.

[12] Women and Equalities Committee (2019). Health and Social Care and LGBT Communities. https://publications.parliament.uk/pa/cm201919/cmselect/cmwomeq/94/9406.htm.

[13] Hafford-Letchfield T (2008). What’s love got to do with it? Developing supportive practices for the expression of sexuality, sexual identity and the intimacy needs of older people. J Care Serv Manag.

[14] Simpson P, Almack K, Walthery P (2018). ‘We treat them all the same’: the attitudes, knowledge and practices of staff concerning old/er lesbian, gay, bisexual and trans residents in care homes. Ageing Soc.

[15] Willis P (2017). Queer, visible, present: the visibility of older LGB adults in long-term care environments. HCS.

[16] Breder K, Bockting W (2023). Social networks of LGBT older adults: An integrative review. Psychol Sex Orientat Gend Divers.

[17] Guasp A (2011). Lesbian, gay and bisexual in later life. https://www.ageuk.org.uk/bp-assets/globalassets/shropshire-telford--wrekin/original-blocks/our-services/useful-links/stonewall-lesbian-gay-and-bisexual-people-in-later-life.pdf.

[18] Fredriksen-Goldsen KI, Kim H-J, Barkan SE (2013). Health disparities among lesbian, gay, and bisexual older adults: results from a population-based study. Am J Public Health.

[19] Savage B, Barringer MN (2021). The (minority) stress of hiding: the effects of LGBT identities and social support on aging adults’ concern about housing. Sociol Spectr.

[20] Ipsos (2021). LGBT+ pride 2021 global survey. https://www.ipsos.com/sites/default/files/ct/news/documents/2021-06/lgbt-pride-2021-global-survey-ipsos.pdf.

[21] Age UK (2020). The story of Age UK’s LGBT+ section. https://www.ageuk.org.uk/discover/2020/02/age-uk-lgbt-plus-section/.

[22] Office for National Statistics (2023). Sexual orientations, england and wales: census 2021. https://www.ons.gov.uk/peoplepopulationandcommunity/culturalidentity/sexuality/bulletins/sexualorientationenglandandwales/census2021#:~:text=straight%20or%20heterosexual%20(89.4%25%20of,in%20both%20England%20and%20Wales).

[23] Office for National Statistics (2023). Gender identity, England and Wales: Census 2021.

[24] Conron KJ, Flores AR (2023). Adult LGBT population in the united states. https://williamsinstitute.law.ucla.edu/publications/adult-lgbt-pop-us/.

[25] Sage (2025). Facts on LGBTQ+ aging. https://www.sageusa.org/resource/facts-on-lgbt-aging/.

[26] Australian Institute of Health and Welfare (2025). Older australians who identify as lesbian, gay, bisexual, transgender or intersex. https://www.aihw.gov.au/reports/older-people/older-australians/contents/population-groups-of-interest/identify-as-lgbti.

[27] Hafford-Letchfield T, Simpson P, Willis PB (2018). Developing inclusive residential care for older lesbian, gay, bisexual and trans (LGBT) people: An evaluation of the Care Home Challenge action research project. Health Soc Care Community.

[28] Jurček A, Downes C, Keogh B (2021). Educating health and social care practitioners on the experiences and needs of older LGBT+ adults: Findings from a systematic review. J Nurs Manag.

[29] Keemink JR, Hammond J, Collins G (2025). The CIRCLE Care Home Guide: A Co‐Designed Resource on LGBTQ+ Inclusion for Care Homes. Health Expect.

[30] Rataj AC, Porter KE, Dugan E (2025). Changing attitudes about LGBTQ+ older adults: *The Gen Silent Survey Project*. Gerontol Geriatr Educ.

[31] Daley AE, Macdonnell JA (2011). Gender, sexuality and the discursive representation of access and equity in health services literature: implications for LGBT communities. Int J Equity Health.

[32] Department of Health (2025). Care and support statutory guidance: issued under the care act 2014. https://www.gov.uk/government/publications/care-act-statutory-guidance/care-and-support-statutory-guidance.

[33] Department of Health & Social Care (2025). Road to recovery: the government’s 2025 mandate to NHS England. https://www.gov.uk/government/publications/road-to-recovery-the-governments-2025-mandate-to-nhs-england/road-to-recovery-the-governments-2025-mandate-to-nhs-england.

[34] Monitor (2015). Moving healthcare close to home: summary. https://assets.publishing.service.gov.uk/media/5a802282ed915d74e33f8a69/moving_healthcare_closer_to_home_summary.pdf.

[35] Chen R, Jiang Y, Li X (2024). Community-based elderly home care services policy and household consumption enhancement: Evidence from China. Int. Rev. Econ. Finance..

[36] Genet N, Boerma WG, Kringos DS (2011). Home care in Europe: a systematic literature review. BMC Health Serv Res.

[37] Palesy D, Jakimowicz S, Saunders C (2018). Home care in Australia: an integrative review. Home Health Care Serv Q.

[38] Schuchman M, Fain M, Cornwell T (2018). The Resurgence of Home-Based Primary Care Models in the United States. Geriatrics (Basel).

[39] Willis P, Raithby M, Maegusuku-Hewett T (2018). “It’s a nice country but it’s not mine”: Exploring the meanings attached to home, rurality and place for older lesbian, gay and bisexual adults. Health Soc Care Community.

[40] Malley J, D’Amico F, Fernandez J-L (2019). What is the relationship between the quality of care experience and quality of life outcomes? Some evidence from long-term home care in England. Soc Sci Med.

[41] Duffy M, Frazzetto G, Staines A (2024). A Scoping Review of Older LGBTI People’s Experiences of Homecare. *SI*.

[42] May JT, Crist JD (2023). Healthcare Worker Perceptions of LGBTQ+ Older Adults: Integrative Review. Clin Nurs Res.

[43] Smith R, Wright T (2021). Older lesbian, gay, bisexual, transgender, queer and intersex peoples’ experiences and perceptions of receiving home care services in the community: A systematic review. Int J Nurs Stud.

[44] Garritty C, Gartlehner G, Nussbaumer-Streit B (2021). Cochrane Rapid Reviews Methods Group offers evidence-informed guidance to conduct rapid reviews. J Clin Epidemiol.

[45] Garritty C, Hamel C, Trivella M (2024). Updated recommendations for the Cochrane rapid review methods guidance for rapid reviews of effectiveness. BMJ.

[46] Moons P, Goossens E, Thompson DR (2021). Rapid reviews: the pros and cons of an accelerated review process. Eur J Cardiovasc Nurs.

[47] Schünemann HJ, Moja L (2015). Reviews: Rapid! Rapid! Rapid! …and systematic. Syst Rev.

[48] Page MJ, McKenzie JE, Bossuyt PM (2021). The PRISMA 2020 statement: an updated guideline for reporting systematic reviews. BMJ.

[49] Stevens A, Hersi M, Garritty C (2025). Rapid review method series: interim guidance for the reporting of rapid reviews. BMJ Evid Based Med.

[50] Shamseer L, Moher D, Clarke M (2015). Preferred reporting items for systematic review and meta-analysis protocols (PRISMA-P) 2015: elaboration and explanation. BMJ.

[51] Nussbaumer-Streit B, Klerings I, Dobrescu AI (2020). Excluding non-English publications from evidence-syntheses did not change conclusions: a meta-epidemiological study. J Clin Epidemiol.

[52] Almack K, King A (2019). Lesbian, Gay, Bisexual, and Trans Aging in a U.K. Context: Critical Observations of Recent Research Literature. Int J Aging Hum Dev.

[53] Westwood S, Willis P, Fish J (2020). Older LGBT+ health inequalities in the UK: setting a research agenda. *J Epidemiol Community Health*.

[54] (2025). Digital scholarship. https://www.zotero.org/.

[55] Harrison R, Jones B, Gardner P (2021). Quality assessment with diverse studies (QuADS): an appraisal tool for methodological and reporting quality in systematic reviews of mixed- or multi-method studies. BMC Health Serv Res.

[56] Keemink JR, Glass D, Dargan AK (2023). The Role of Adult Social Care in the Prevention of Intensive Health and Care Needs: A Scoping Review. Journal of Long Term Care.

[57] Thomas J, Harden A (2008). Methods for the thematic synthesis of qualitative research in systematic reviews. BMC Med Res Methodol.

[58] Gough D, Thomas J, Oliver S (2012). Clarifying differences between review designs and methods. Syst Rev.

[59] Campbell M, McKenzie JE, Sowden A (2020). Synthesis without meta-analysis (SWiM) in systematic reviews: reporting guideline. BMJ.

[60] Guyatt G, Oxman AD, Akl EA (2011). GRADE guidelines: 1. Introduction-GRADE evidence profiles and summary of findings tables. J Clin Epidemiol.

