# Supplementary material for: Evidence on the preparedness and practice needs of the home care workforce to support older LGBTQ+ people: a rapid review protocol
Source: BMJ Open. 2026 Feb 2;16(2):e110207. doi: 10.1136/bmjopen-2025-110207 (PMC12878354; doi:10.1136/bmjopen-2025-110207)
Supplement: online supplemental file 1 [file bmjopen-16-2-s001.docx]

**Appendix 1**
Completed PRISMA-P checklist

| Section & topic | Item no. | Checklist item |
| --- | --- | --- |
| **Administrative information** | | |
| Title: |  | Protocol for a rapid review of the evidence on the preparedness and practice needs of the home care workforce to support older LGBTQ+ people. |
| Identification | 1a | We have noted that this is a protocol in the title, the abstract, and the main body of the paper (p.5). |
| Update | 1b | Not applicable |
| Registration | 2 | PROSPERO CRD420251038242, mentioned in the abstract and on p.6 in the methods section. |
| Authors: |  | Keemink, J. R., Stander, W. J., Thomas, B., & Willis, P. |
| Contact | 3a | Reported on title page |
| Contributions | 3b | Reported on p.14 |
| Amendments | 4 | This protocol was updated after peer review for journal publication. |
| Support: |  |  |
| Sources | 5a | Reported on p.14 |
| Sponsor | 5b | Reported on p.14 |
| Role of sponsor or funder | 5c | Reported on p.14 |
| **Introduction** | | |
| Rationale | 6 | Reported on p.3,4&5 |
| Objectives | 7 | Reported on p.7 |
| **Methods** | | |
| Eligibility criteria | 8 | Reported on p.7&8 |
| Information sources | 9 | Reported on p.9 |
| Search strategy | 10 | Reported on p.9&10 |
| Study records: |  |  |
| Data management | 11a | Reported on p.10&11 |
| Selection process | 11b | Reported on p.10&11 |
| Data collection process | 11c | Reported on p.11 |
| Data items | 12 | Reported on p.7 |
| Outcomes and prioritisation | 13 | Reported on p.7 |
| Risk of bias in individual studies | 14 | Reported on p.11 |
| Data synthesis | 15a | Reported on p.12 |
|  | 15b | Reported on p.12 |
|  | 15c | Not applicable |
|  | 15d | Reported on p.12 |
| Meta-bias(es) | 16 | Not applicable |
| Confidence in cumulative evidence | 17 | Reported on p.12 |
