# Supplementary material for: Evidence on the preparedness and practice needs of the home care workforce to support older LGBTQ+ people: a rapid review protocol
Source: BMJ Open. 2026 Feb 2;16(2):e110207. doi: 10.1136/bmjopen-2025-110207 (PMC12878354; doi:10.1136/bmjopen-2025-110207)
Supplement: Supplementary file 2 [file bmjopen-2025-110207.reviewer_comments.pdf]

PEER REVIEW HISTORY

BMJ Open publishes all reviews undertaken for accepted manuscripts. Reviewers are asked to complete a checklist review form and are provided with free text boxes to elaborate on their assessment. These free text comments are reproduced below.

ARTICLE DETAILS

Title (Provisional)

Protocol for a rapid review of the evidence on the preparedness and practice needs of the home care workforce to support older LGBTQ+ people.

Authors

Keemink, Jolie R.; Stander, Willem J.; Thomas, Benjamin; Willis, Paul

---

VERSION 1 - REVIEW

**Reviewer**	**1**
**Name**	**Morris, Meg**
**Affiliation**	**La Trobe Univ, Room 423 HS3**
**Date**	**29-Oct-2025**
**COI**	**None**

This scholarly and well-written protocol article aims to analyze what is known

about the preparedness and practice needs of the care workforce who work in the home to support older LGBTQ+ people to maintain quality of life.

1. Please remove the following sentence from the strength and limitations section: "We followed the Preferred Reporting Items for Systematic Reviews and MetaAnalysis Protocols (PRISMA-P) for this protocol and have included a completed PRISMA-P checklist."

2. In the Introduction please remove any words or phrases that could be considered to be non-evidence-based such as "a landscape of precarious interpretations"

3. In the Introduction please operationally define what is meant by "care workforce" and describe the profile of the care workforce in home based care.

4. In the Introduction please give references that provide evidence for the statement that care service providers "hold discriminatory assumptions rooted in ageism, heteronormativity and cisnormativity" and define cisnormativity in brackets.

5. In the Introduction please give US and European data and data from other countries across the globe alongside the statement that "at least 3.3% of the population in England

and Wales identifies as LGB, and 0.5% with a gender different from their registered birth sex (~2.6 million people)

6. In the Introduction please have more of a global perspective rather than focussing only on the UK.

7. In the Methods please give a reference when you mention the Cochrane recommendations.

8. When you say all designs will be included, please provide a list of the range of designs that this refers to.

9. Please justify why an inclusion criteria is published in English and in your manuscript please discuss the limitations to generalisability this creates.

10. Please change the sentence to say the search will be carried out in August 2025 to November 2025 given that August has already passed.

11. Please explain what Zotero refers to and give a reference.

12. Will you be using Covidence or databases for this review or are you solely relying on excel spreadsheets?

13. The Discussion section in this manuscript is very short and tends to repeat what was said earlier. Please can you edit it to compare and contrast your methods with other studies of this type across the world and synthesize the impact this important study will have.

14. Please consider broadening your search strategy to cite a wider range of care workforce domains to obtain a broader literature - some suggestions that could be considered are:

Anza-Ramirez, C., Miranda, J. J., Armocida, B., Correia, J. C., Van Spall, H. G. C., Beran, D., . . . GBD 2019 Acute and Chronic Care Collaborators. (2025). Characterising acute and chronic care needs: insights from the Global Burden of Disease Study 2019. Nature Communications, 16, 1-36. doi:10.1038/s41467-025-56910-

Morris, M. E., Brusco, N. K., McAleer, R., Billett, S., Brophy, L., Bryant, R., . . . Blackberry, I. (2023). Professional care workforce: a rapid review of evidence supporting methods of recruitment, retention, safety, and education. Human Resources for Health, 21, 34 pages. doi:10.1186/s12960-023-00879-5

Thwaites, C., McKercher, J. P., Fetherstonhaugh, D., Blackberry, I., Gilmartin-Thomas, J. F. -M., Taylor, N. F. (2023). Factors Impacting Retention of Aged Care Workers: A Systematic Review. Healthcare (Switzerland), 11(23), 24 pages. doi:10.3390/healthcare11233008

**Reviewer**	**2**
**Name**	**Braeckman, Lutgart**

Affiliation **Universiteit Gent Vakgroep Maatschappelijke Gezondheidkunde**

Date **09-Nov-2025**

COI **None**

This paper presents the protocol for a rapid review to synthesise existing evidence on the preparedness and practice needs of the home care workforce to care for older LGBTQ+ people. The protocol is written concisely and overall provides a clear description of the methods to review the literature and the analysis that will be done (has been done). The idea of studying more in depth and prospectively the preparedness and needs of the home care workforce to support older LGBTQ+ people is interesting and relevant. Nevertheless, there are some minor points that merit more attention.

- The protocol paper should report planned or ongoing studies. This protocol for a rapid review was submitted 1st September 2025, the search was carried out in August 2025 (p 8, line 8). The current review is part of a larger funded study and results will provide suggestions for later stages in the project (p 5, line 40-42). So, I expect the draft search strategy was already finalized when the paper was submitted?

- The intern guidance for rapid reviews (PRISMA-RR, Stevens et al., 2024; p 5 line 51) is still under construction?

- P 4 line 3-9 : numbers are given such as 3.3% identifies as LGB and 0.5 % with a gender different from their registered birth sex. Of those an estimated 1 million people are over 60 …Footnote 1. I find the figures difficult to interpret and the footnote has ended up in the text (in my version).

- Only in the PICO statement (p 6) does it become clear what you mean by the older LGBTQ+ population (e.g. > 60 years; where on p 4, line 8, you write somewhat suggestively "1 million people over 60") and by "being prepared". I would suggest to also mention this in the introduction and explain the reasons for choosing these definitions.

- P5 : you mention the advantage of time efficiency and rapid collection of evidence to conduct a rapid review. Can you elaborate on possible disadvantages?

- P 11 : Public involvement. One older LGBTQ+ person contributed to the funding application, … and method for the rapid review. I would like to suggest involving more people from the LGBTQ+ community in the rest of the project, as each gender identity has specific needs.

VERSION 1 - AUTHOR RESPONSE

Reviewer 1

Comment	Author response
Please remove the following sentence from the strength and limitations section: "We followed the Preferred Reporting Items for Systematic Reviews and MetaAnalysis Protocols (PRISMA-P) for this protocol and have included a completed PRISMA-P checklist."	We have now removed this.
In the Introduction please remove any words or phrases that could be considered to be non-evidence-based such as "a landscape of precarious interpretations"	We have endeavoured to remove all language that could be considered non-evidence-based. Please see tracked changes in introduction.
In the Introduction please operationally define what is meant by "care workforce" and describe the profile of the care workforce in home based care.	We have now included a definition of what is meant by the term 'adult social care' used in England and Wales. Please see page 3.
In the Introduction please give references that provide evidence for the statement that care service providers "hold discriminatory assumptions rooted in ageism, heteronormativity and cisnormativity" and define cisnormativity in brackets.	We have now provided references, and included a definition of cisnormativity on page 3.
In the Introduction please give US and European data and data from other countries across the globe alongside the statement that "at least 3.3% of the population in England and Wales identifies as LGB, and 0.5% with a gender different from their registered birth sex (~2.6 million people)	We have updated this section to reflect the nuances on data on older LGBTQ+ people worldwide. See page 4.
In the Introduction please have more of a global perspective rather than focussing only on the UK.	We have removed most wording that suggests a UK-only perspective and have added literature and context providing a global perspective.
In the Methods please give a reference when you mention the Cochrane recommendations.	Many thanks for highlighting this omission. The reference has now been added both in-text and in the reference section.
When you say all designs will be included, please provide a list of the range of designs that this refers to.	We have now specified this on page 8.
Please justify why an inclusion criteria is published in English and in your manuscript please discuss the	We have now provided a justification for this on page 8.

limitations to generalisability this creates.	
Please change the sentence to say the search will be carried out in August 2025 to November 2025 given that August has already passed.	We have updated this as per your suggestion, see page 9.
Please explain what Zotero refers to and give a reference.	We have now added this on page 11.
Will you be using Covidence or databases for this review or are you solely relying on excel spreadsheets?	We will be exclusively relying on Excel spreadsheets. It was anticipated that the included body of research was small enough to manage in Excel, and this was freely available to the research team.
The Discussion section in this manuscript is very short and tends to repeat what was said earlier. Please can you edit it to compare and contrast your methods with other studies of this type across the world and synthesize the impact this important study will have.	On the advice of the editor, we have removed the Results and Discussion section, as this is a protocol. We will make sure to use your recommendations of contrasting methods and synthesising impact in the Discussion section of the manuscript of the actual rapid review.
Please consider broadening your search strategy to cite a wider range of care workforce domains to obtain a broader literature - some suggestions that could be considered are:<br>Anza-Ramirez, C., Miranda, J. J., Armocida, B., Correia, J. C., Van Spall, H. G. C., Beran, D., . . . GBD 2019 Acute and Chronic Care Collaborators. (2025). Characterising acute and chronic care needs: insights from the Global Burden of Disease Study 2019. Nature Communications, 16, 1-36. doi:10.1038/s41467-025-56910-<br><br>Morris, M. E., Brusco, N. K., McAleer, R., Billett, S., Brophy, L., Bryant, R., . . . Blackberry, I. (2023). Professional care workforce: a rapid review of evidence supporting methods of recruitment, retention, safety, and education. Human Resources for Health, 21, 34 pages. doi:10.1186/s12960-023-00879-5<br><br>Thwaites, C., McKercher, J. P., Fetherstonhaugh, D., Blackberry, I., Gilmartin-Thomas, J. F. -M., Taylor, N. F.	Many thanks for these suggestions. This review deliberately focuses exclusively on the home care workforce as they are an under researched part of the care workforce. Broader reviews including literature on the entire long-term or aged care workforce already exist (e.g. Caceres et al., 2020; May & Christ, 2023), but home care is often an afterthought in these broader reviews. With the current review, we aim to highlight the specific experiences and challenges in relation to home care (for reasons detailed in the Introduction section), which will also help identity important research gaps in relation to LGBTQ+ inclusion in home care and inform later elements of the wider study on LGBTQ+ inclusion in home care that this review is a part of.

(2023). Factors Impacting Retention of Aged Care Workers: A Systematic Review. Healthcare (Switzerland), 11(23), 24 pages. doi:10.3390/healthcare11233008	

Reviewer 2

Comment	Author response
The protocol paper should report planned or ongoing studies. This protocol for a rapid review was submitted 1st September 2025, the search was carried out in August 2025 (p 8, line 8). The current review is part of a larger funded study and results will provide suggestions for later stages in the project (p 5, line 40-42). So, I expect the draft search strategy was already finalized when the paper was submitted?	Apologies for any confusion around this. The pilot search was carried out in August and the full search in September. However the search was ongoing as further papers and grey literature were identified between August and November. As per suggestion from reviewer 1, we have changed the search period to 'between August and November 2025', see page 9.
The intern guidance for rapid reviews (PRISMA-RR, Stevens et al., 2024; p 5 line 51) is still under construction?	Whilst the Stevens et al. paper provides interim guidelines, it does include suggestions for a minimum set of items for rapid reviews, and these have informed our reporting.
P 4 line 3-9 : numbers are given such as 3.3% identifies as LGB and 0.5 % with a gender different from their registered birth sex. Of those an estimated 1 million people are over 60 …Footnote 1. I find the figures difficult to interpret and the footnote has ended up in the text (in my version).	Apologies for the confusion. Reviewer 1 also commented on this section and we have revised it to make it clearer and more internationally focused, see page 4. The footnote has now been removed.
Only in the PICO statement (p 6) does it become clear what you mean by the older LGBTQ+ population (e.g. > 60 years; where on p 4, line 8, you write somewhat suggestively "1 million people over 60") and by "being prepared". I would suggest to also mention this in the introduction and explain the reasons for choosing these definitions.	Thank you for pointing this out. We have now mentioned this on p.3 and explained that this definition was chosen in consultation with the study's public advisory group.
P5 : you mention the advantage of time efficiency and rapid collection of evidence to conduct a rapid review. Can you elaborate on possible disadvantages?	We have now provided more details on limitation on page 6 and page 8.

P 11 : Public involvement. One older LGBTQ+ person contributed to the funding application, ... and method for the rapid review. I would like to suggest involving more people from the LGBTQ+ community in the rest of the project, as each gender identity has specific needs.	Thank you for this suggestion. As mentioned in the manuscript, our public advisory group will feed into later stages of the review, including interpretation of findings. This group includes five older LGBTQ+ people, including a trans person, and all bring experience of receiving or working in adult social care.

VERSION 2 - REVIEW

Reviewer 2

Name Braeckman, Lutgart

Affiliation Universiteit Gent Vakgroep Maatschappelijke Gezondheidkunde

Date 17-Dec-2025

COI

The authors have adequately answered my questions and explained their methodology more clearly.

There are still a few minor issues that need to be resolved.

P 4 The word ‘data’ is singular, so the corresponding verb should be in the third person singular.

P 7 PICO statement. You already gave the definition of preparedness in the Introduction.

P 9 typo line 41 : workforce support organisations

P 11 one reviewer (not reviewers) : line 15 & line 51
